# Geographic and ecological drivers of contaminants and nutrients in commercial fish species

**DOI:** 10.1016/j.scitotenv.2025.179786

**Published:** 2025-05-29

**Authors:** Vivien F. Taylor, Kate L. Buckman, Celia Y. Chen, Jana Kraft

**Affiliations:** a Department of Earth Sciences, Dartmouth College, Hanover, NH 03755, United States of America; b Connecticut River Conservancy, Greenfield, MA 01301, United States of America; c Department of Biological Sciences, Dartmouth College, Hanover, NH 03755, United States of America; d Department of Animal and Veterinary Sciences, University of Vermont, Burlington, VT 05405, United States of America

**Keywords:** Mercury, Selenium, Lipid content, Stable isotope, Location, Commercial fish

## Abstract

Commercial marine fish are a major source of exposure to harmful compounds such as methylmercury (MeHg) but are also an important source of nutrients including selenium (Se) and omega-3 polyunsaturated fatty acids (ω-3). Safe eating guidelines seek to provide information to minimize the risk of contaminant exposure, while promoting the nutritional benefits of eating fish, yet information on the patterns and drivers of contaminants and nutrients are scarce, particularly within and across populations of commercially harvested marine fish species. This study assessed total mercury (Hg), Se and total lipids, along with subsets of MeHg and ω-3, in muscle tissue of six species of fish (Atlantic cod, Atlantic herring, red hake, silver hake, little skates and spiny dogfish) caught in the Gulf of Maine, the largest coastal fishery in the U.S. Fish species, as well as size and catch location (inshore vs. offshore) had a significant effect on concentrations of Hg and lipids, whereas Se concentrations varied little, except in skate. Higher length-adjusted concentrations of Hg and lower concentrations of lipids were found inshore for Atlantic cod, whereas the reverse was true for Atlantic herring, and Hg was also higher in offshore spiny dogfish. Stable isotope analysis and differences in catch timing suggest these patterns are due to seasonal and locational differences in plankton community compositions and in prey availability to pelagic and demersal fish. These findings reveal that local environment plays an important role in determining both nutrient and contaminant load, and points to the effects of environmental change on the risk-inducing and beneficial qualities of commercially caught fish.

## Graphical Abstract

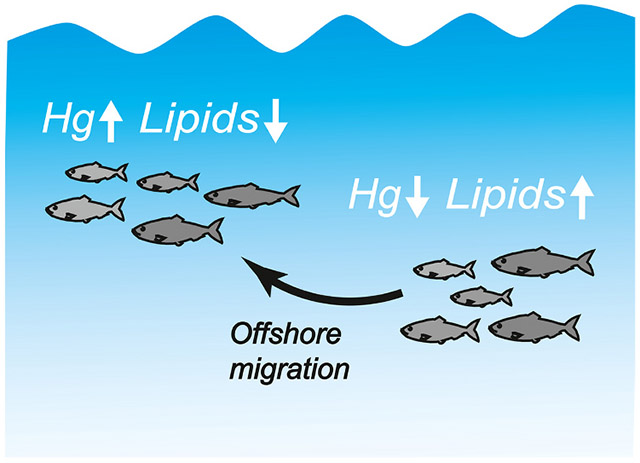

## 1. Introduction

Fish consumption is a source of both beneficial nutrients and harmful contaminants, which has made it difficult to provide clear health advice to consumers. Fish consumption guidelines exist for all coastal waters in the U.S., with mercury (Hg) being the primary cause. Mercury in its organic form, methylmercury (MeHg), bioaccumulates and biomagnifies in aquatic food webs. Methylmercury is a potent neurotoxin, and fish tissue concentrations in large and high-trophic level fish often exceed the criteria of 0.3 mg kg^−1^ (U.S. EPA, 2001) or the species-specific maximum levels (0.5 mg kg^−1^ for most species, 0.3 mg kg^−1^ for selected species including cod and herring and 1.0 for some species including sharks) (European Commision, 2023) established for reducing risk to human health. These criteria provide useful guidelines, though specific intake recommendations vary with body mass, portion size and consumption rate (FAO, 2024). Public health advice and to some extent confusion about Hg exposure has led to consumer concerns over eating fish, sometimes resulting in a reluctance to consume seafood (Oken et al., 2003).

Fish are an important source of nutrients, serving as the major dietary source of omega-3 polyunsaturated fatty acids (ω3), which are essential nutrients for human development and health (Gribble et al., 2015; Mahaffey, 2004; Swanson et al., 2012), and an important source of the trace element selenium (Ralston and Raymond, 2010), which is thought to play a role in reducing risk of cancer, cardiovascular disease, cognitive decline, and thyroid disease (Lei et al., 2024). These nutrients may also be protective to some of the harmful effects of Hg: the ω3s, eicosapentaenoic acid (EPA) and docosahexaenoic acid (DHA), have been shown to compete with the deleterious effects of Hg on neurological development (Oken et al., 2008). There is also evidence that Se intake has a countering effect on Hg exposure, and a molar ratio >1:1 Se: Hg in fish has been suggested to reduce the toxic effects of Hg (Burger and Gochfeld, 2013; Ralston and Raymond, 2010), though the use of this ratio as a measure of health protectivity is contentious (Gerson et al., 2020). Public health advice around fish consumption can therefore be contradictory by encouraging higher nutrient intake, particularly among children and pregnant women, while also discouraging consumption to reduce exposure to contaminants. The potentially competing health effects of Hg, ω-3 s and Se drive the need for assessments of concurrent levels of contaminants and nutrients across species and within the same species and populations of fish (Gribble et al., 2015; Le Croizier et al., 2016). Furthermore, rapid environmental changes in coastal environments have been suggested to increase fish Hg concentrations (Schartup et al., 2019) and reduce beneficial fatty acids in fish (Bi et al., 2020; Hixson and Arts, 2016). Insight into the patterns and environmental drivers of nutrients and contaminants are important to assessing trends in the nutritional value of fish.

The Gulf of Maine is an important fishing ground for both domestic US and international fisheries and aquaculture. A large percentage of commercial catches go towards human consumption, while other fisheries support baitfish and fish meal industries. Commercially important species commonly found and fished in the Gulf of Maine that were utilized in this study include cod (*Gadus morhua*), red hake (*Urophycis chuss*), silver hake (*Merluccius bilinearis*), Atlantic herring (*Clupea harengus*), little skates (*Leucoraja erinacea*), and spiny dogfish (*Squalus acanthias*). Cod, hake, herring, and dogfish are all commonly consumed by humans although the US commercial landings of dogfish are typically intended for the European market (NOAA, 2024). Little skates are often utilized for bait. In these commercially important fish species, we aim to understand the within and between species variation of fillet tissue concentrations of both detrimental contaminants and beneficial nutrients. This study addresses three questions: 1) How do the concentrations and drivers of Hg, Se, and lipids vary by fish species and habitat in the Gulf of Maine? 2) How are concentrations of Hg, Se, and lipids within fish species affected by fish length and location? and 3) What are the ecological drivers of Hg, Se, and lipid concentrations in fish?

## 2. Methods

### 2.1. Sample collection & processing

**Fig. 1.**
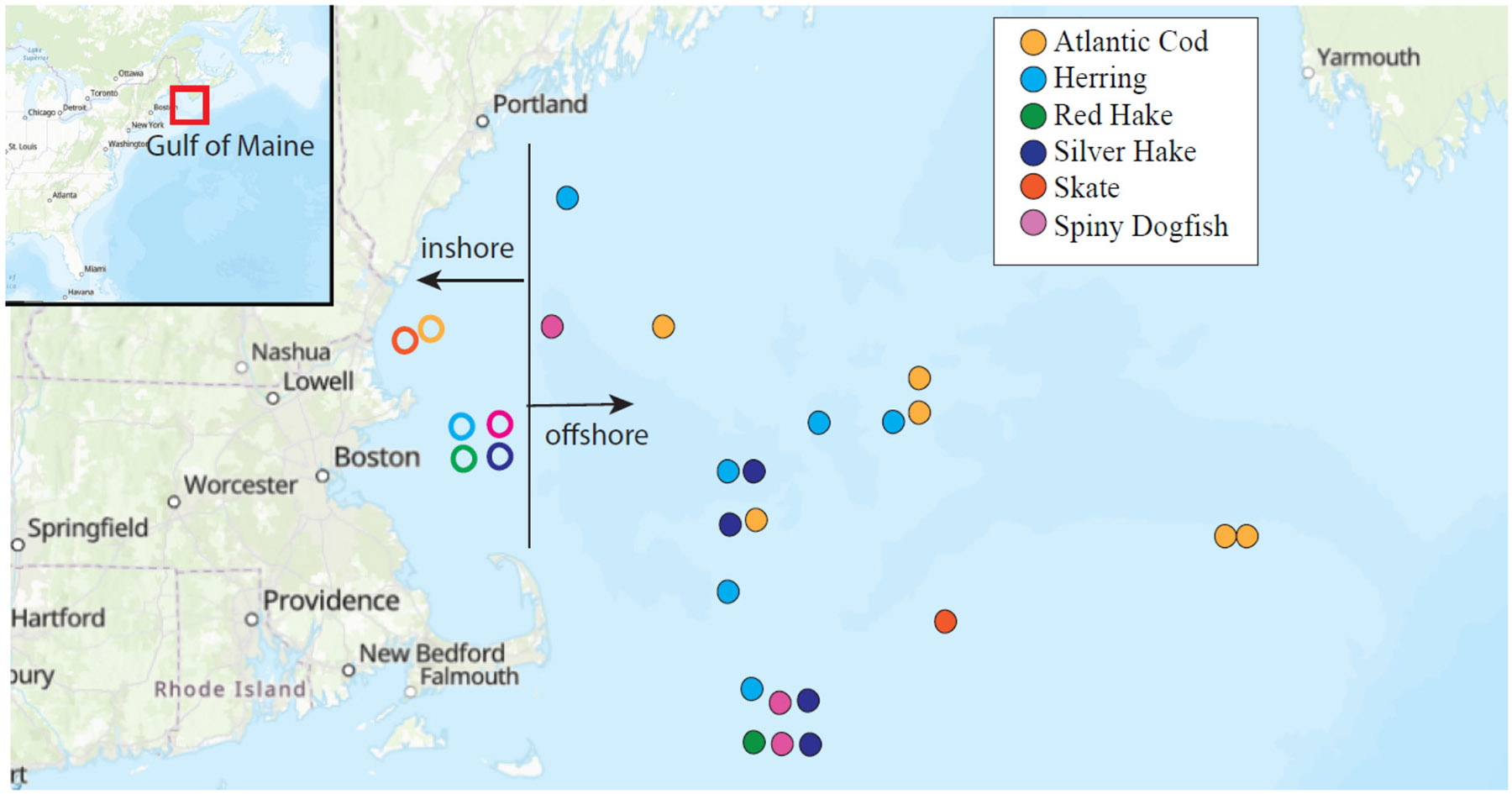
Catch locations of fish in the Gulf of Maine.

Fish were collected with assistance from Capt. David Goethel of the *Ellen Diane*, the Yankee Fisherman’s Cooperative (Seabrook NH), and the National Marine Fisheries Service (Woods Hole, MA) through their biennial demersal bottom trawl survey in the Gulf of Maine. We categorized catch location as either “inshore” or “offshore”, with inshore fish collected in shallower waters (<50 m) west of Jeffrey’s Ledge, whereas offshore fish were collected in deeper waters east of that (Fig. 1).

Inshore cod (*n* = 17) and skates (*n* = 29) were collected 26 May 2018, inshore red hake (*n* = 32) and silver hake (*n* = 33), dogfish (*n* = 20), and herring (*n* = 16) were collected 10 July 2018, and offshore fish of all species were collected 12 Oct through 5 Nov 2018 from various locations (cod: *n* = 17; skate: *n* = 20; red hake: *n* = 30; silver hake: *n* = 31; dogfish: *n* = 30; herring: *n* = 19). All fish were collected using trawls. Fish were weighed, measured (total length), and sex determined (when possible) prior to tissue processing. Inshore fish were kept on ice from collection until processing in the lab, which occurred within 24 h of collection. Offshore fish collected during the NMFS fall bottom trawl survey were frozen at sea and partially thawed at a later date for processing. In the lab, fish were filleted and subsamples of the right fillet from the upper anterior portion (skin off) were taken for total metal and total lipid analysis and frozen. For skates, width from wing tip to wing tip was measured, and tissue for analysis was dissected from the right wing close to the body. Samples of individual fish were analyzed, with the exception of very small individuals, where it was necessary to combine both fillets to obtain enough tissue for both trace metal and total lipid analysis.

### 2.2. Analytical

#### 2.2.1. Metals

Samples intended for total metal analysis were further processed in a trace metal clean room, where they were weighed wet, freeze-dried (Labconco Freezone 4.0), and re-weighed for dry weight. The dry tissue was snipped into fine pieces using acid-rinsed ceramic scissors and ground. Approximately 0.05–0.1 g of the skin off muscle tissue was microwave digested in 9:1 trace metal grade nitric: hydrochloric acid. For lipid-rich tissues an addition of 0.3 ml Optima grade hydrogen peroxide was added after the initial microwave digest. The digest was diluted to 10 % acid by weight with ultra-pure water and submitted to the Dartmouth Trace Element Analysis Core for total metal concentration analysis by ICP-MS. Recoveries of Hg in standard reference materials were 95 % ± 9 (mean ± standard deviation) for DORM-4 Fish Protein (*n* = 17) (National Research Council of Canada), 104 % ± 12 for TORT-3 Lobster Hepatopancreas (*n* = 12) (National Research Council of Canada), and 119 % ± 31 for NIST 2976 mussel (*n* = 6) (National Institute of Standards and Technology). Percent recovery for Se was 98 % ±9 for DORM 4 (*n* = 17), and 92 % ±6 for TORT-3 (*n* = 12), and 103 % ± 3 for NIST mussel (*n* = 6). Eighteen duplicate samples were run, with relative percent difference (RPD) of 10 ± 8 % for total Hg and 6 ± 6 % for Se.

Six individuals from each species and location (inshore/offshore), spanning a range of fish lengths were chosen for MeHg speciation analysis. MeHg analysis was conducted at the Dartmouth TEA Core by species-specific isotope dilution ICP-MS using a MERX-M automated methylmercury analyzer coupled to an ICP-MS (Agilent 7900) (Taylor et al., 2011, 2008). Percent recovery for DORM-4 was 88 % (*n* = 2), spike recovery average was 97 % (*n* = 2) and average relative percent difference for duplicates was 1 % (*n* = 2).

#### 2.2.2. Stable isotopes (SI)

Freeze dried, homogenous tissue samples for δ^13^C and δ^15^N analysis were prepared in tin capsules and sent to the UC Davis Stable Isotope Facility for analysis. Whole individual δ^13^C and δ^15^N isotope values were reported relative to international standards (Vienna PeeDee Belemnite for C, atmospheric nitrogen for N) as $\delta \;X({\%}_{0})=([\text{Rsample}∕\text{Rstandard}]-1)\;x\;1000$, where X is either ^13^C or ^15^N and R is the ratio of ^13^C/^12^C or ^15^N/^14^N, respectively. Analytical precision for both δ^13^C and δ^15^N was 0.5 ‰. For herring and dogfish, which have a high lipid content (>15%;C:N>4), δClipid free13 was calculated according to Logan et al., 2008:

δClipid free13=δ13Cbulk−4.763+4.401⋆ln(C:N)

#### 2.2.3. Lipids

Analysis of total lipids was conducted on all the collected fish while fatty acid profiles were determined on a subset. Approximately 5 g of wet tissue were weighed and homogenized in a blender then subject to lipid extraction by a rapid technique using an acetone/ethyl acetate solvent mixture as detailed in (Tölgyessy and Miháliková, 2016). A subset of 12 samples were analyzed by the standard “Folch” extraction and purification method (Folch et al., 1957) for comparison. Lipid content were also examined by the C:N ratio, a proxy for lipid concentration based on the negligible nitrogen (N) content in lipids relative to carbohydrates and proteins (Fagan et al., 2011; Logan et al., 2008; Post et al., 2007). For fatty acid analysis, subsamples of muscle tissue from three individuals from each species and location were sent to NJ Feed Labs (Trenton NJ) to be analyzed by gas chromatography following ether extraction. For two skate samples it was necessary to pool tissue from two similarly sized individuals in order to achieve enough sample mass to meet the laboratory requirements.

#### 2.2.4. SI and fatty acids (FA)

Food source and trophic status across species and catch locations were investigated by a scatterplot of δ^13^C against δ^15^N. The relationship between SI and Hg, Se, and total lipid concentrations for all fish in the study were explored by linear regression.

FA markers were evaluated on muscles tissues, which is influenced by dietary FA’s (Bell et al., 1992, 1995, Budge et al. 2022). Individual FA’s were evaluated as the % of total FA; those with a mean value <0.1 % were removed from the dataset, and the remainder renormalized. Due to the small number of samples and large number of variables, the FA data were analyzed by principal components analysis (PCA) to assess FA’s highly correlated with the principal components, and the associated spread of the samples. The large number of FA’s in the initial analysis was reduced by selecting prevalent individual PUFA’s: EPA (20:5ω3) and DHA (22:6 ω 3), which are indicative of marine phytoplankton (Budge and Parrish, 1998; Dalsgaard et al., 2003) as well as groups of FA’s established as markers: calanoid copepods (20:1, 22:1) (Budge et al., 2022; Copeman et al., 2020; Lee et al., 2006), bivalves (20:1n-7) (Birkely et al., 2003; Budge et al., 2022); benthic invertebrates (n-6 FA) (Budge et al., 2022).

### 2.3. Statistical analysis

Analytical results for Hg, Se, MeHg, and PUFA on a dry weight basis were converted to wet weight-based concentrations using the dry:wet ratio established for each individual when processing the tissue for trace metal analyses (mean = 0.2). Additionally, all concentration data were log10 transformed prior to statistical analyses performed using JMP Pro (SAS) to normalize the data. Initially, multivariate regressions were performed across all fish to assess the influence of habitat, size, location and SI on concentrations of Hg, Se, and lipids. ANOVA (analysis of variance) followed by Tukey’s HSD was used to assess whether there were differences in concentrations between species for Hg, Se and total lipids. Relationships between MeHg and total Hg for all species combined were examined using linear regression as were relationships between total lipids and ω3 and total fatty acid concentration for all samples combined.

As length is known to relate to tissue concentrations of metals and lipids for at least some fish species, ANCOVA (Analysis of Covariance) was utilized to examine relationships between length, catch location and tissue concentrations for Hg, Se, total lipids, δ^13^C. Briefly, we ran a standard least squares model with inshore/offshore catch location, length, and length x catch location as model effects. If there was no significant interaction term, the interaction was removed from the model and the model re-run for the effect of location accounting for the influence of length on concentration. The model was run independently for each species.

## 3. Results and discussion

### 3.1. Concentrations of Hg, Se, and lipids across fish species

**Fig. 2.**
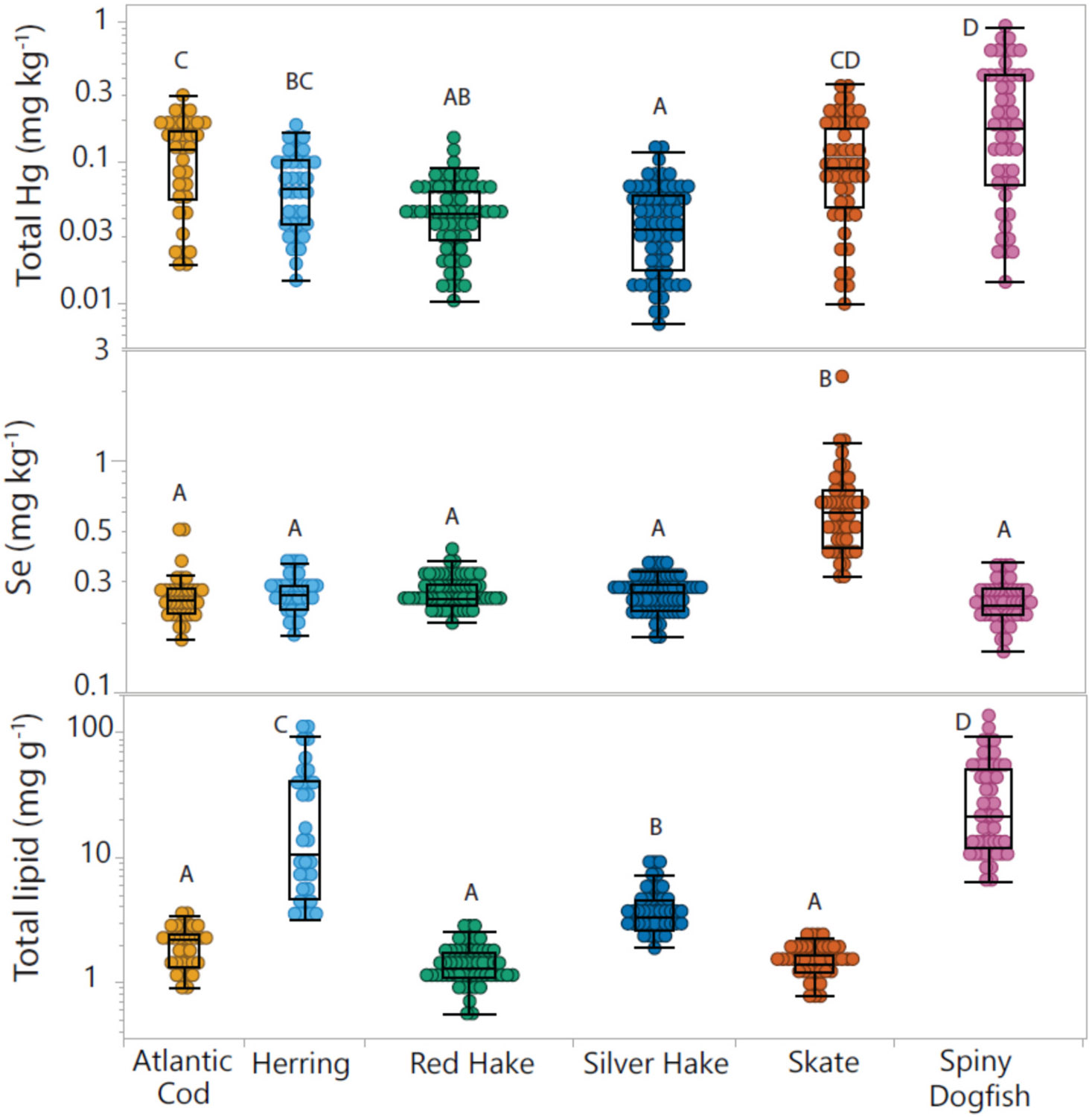
Concentrations of Hg (mg kg^−1^), Se (mg kg^−1^), and total lipids (mg/g) in six fish species (all locations) from the Gulf of Maine. Concentrations in fish species not connected by the same letter are significantly different by Tukey’s post hoc (*p* < 0.05).

Concentrations of Hg, Se and total lipids were first explored across species (Fig. 2, Table 1S). As expected, concentrations of MeHg in a subset of samples (*n* = 36; Table 2S) were strongly correlated with THg concentration (R^2^ = 0.99, *p* < 0.0001) across all species combined (Fig. 1S). Concentrations of Hg in these fish were mostly below the 0.3 ppm EPA criterion (U.S. EPA, 2001), with only the largest dogfish (*n* = 17; 35 % of samples), as well as the largest cod (*n* = 1) and skate (*n* = 2) exceeding it. It is noted that consumption is based on intake rate, and a 227 g portion of the dogfish with the highest Hg concentration (0.89 mg kg^−1^) could be safely consumed ~ once per month by an 80 kg adult according to the U.S. standard (U.S. EPA, 2000). Concentrations of Se were also low, with more elevated concentrations in skate (Fig. 2). Much of the interest in Se concentrations in fish comes from the assertion the Se has a protective effect against the toxic effects of Hg (Ralston et al., 2016; Ralston and Raymond, 2010). In Gulf of Maine fish, molar Se:Hg ratios were driven inversely by Hg concentrations in all species and were above a value of 1, which is deemed protective (Ralston et al., 2016), with the exception of four individual dogfish with the highest Hg levels (Fig. 2S, Table 1S).

**Fig. 3.**
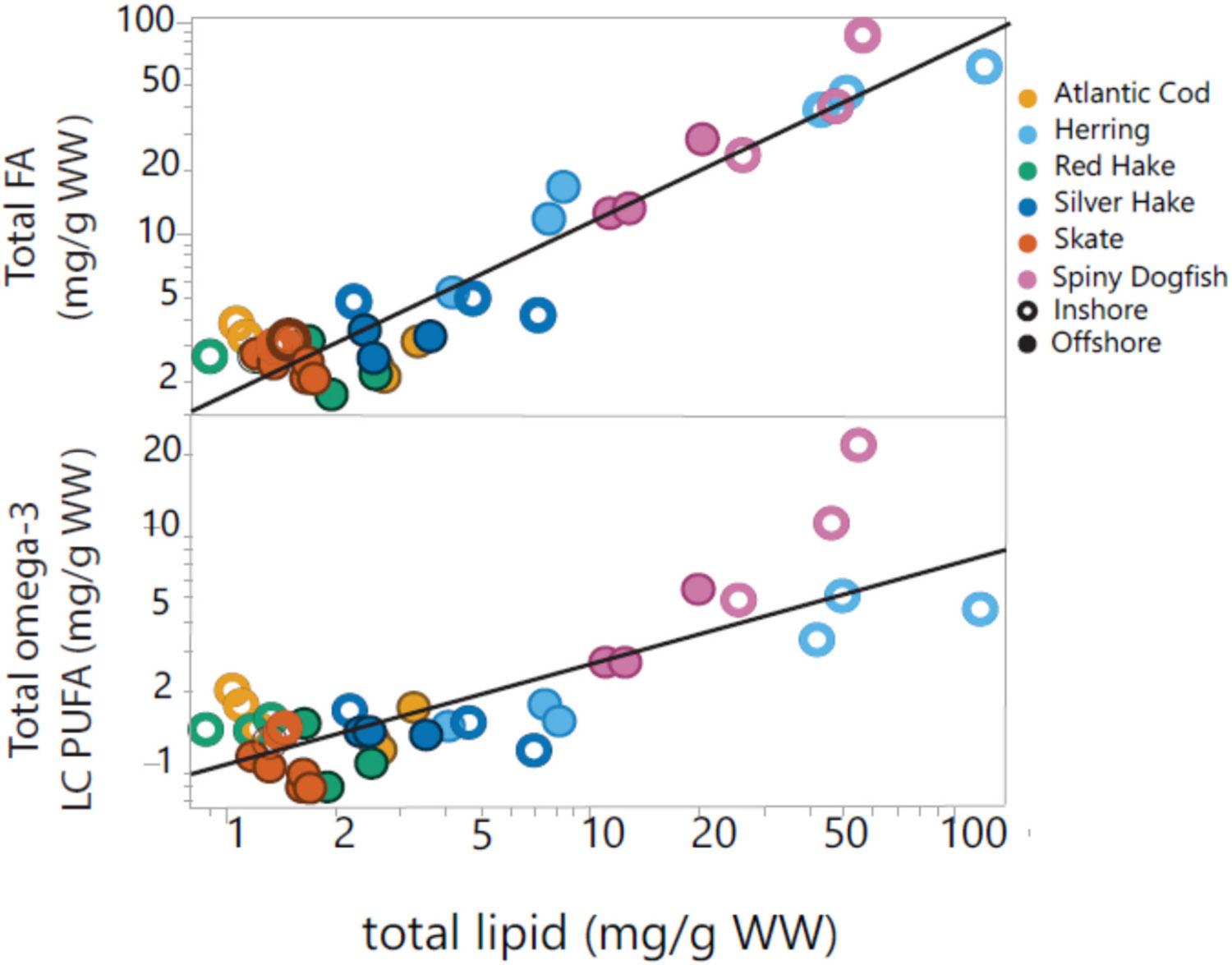
Total lipid concentrations (mg/g wet weight) in fish versus total fatty acid (FA) concentrations (mg/g wet weight) (R^2^ = 0.89) and omega-3 long chain polyunsaturated fatty acid (LC PUFA) concentration (mg/g wet weight) (R^2^ = 0.70) in six fish species from the Gulf of Maine. Fish from the offshore regions are symbolized by solid circles and those from inshore regions are symbolized by open circles. Species are denoted by colors in the legend.

Lipid contents, as expected, were significantly greater in spiny dogfish and herring, than in leaner fish (hake, cod, and skate) (Fig. 2). Lipid concentrations were analyzed by a rapid extraction method in this study (Tölgyessy and Miháliková, 2016), and concentrations were lower than expected for lean fish species (hake, cod and skate), suggesting not all lipid fractions were extracted (Jangaard et al., 1967). This was confirmed by re-analyzing a small subset of samples by the classic Soxhlet extraction method (Folch et al., 1957), which found reasonable agreement between the two methods for high lipid samples, but much lower concentrations in lean fish by the rapid method in (Table 3S). Despite low recoveries of the rapid method, the trends across species were corroborated by C:N ratio (Figs. 5S and 6S), a proxy for lipid content (Fagan et al., 2011). For a subset of samples analyzed for FA composition (*n* = 36; Table 3S), a positive relationship was observed between total lipid and total FA concentrations (R^2^ = 0.89, *p* < 0.0001), and a slightly weaker relationship between total lipids and omega-3 (EPA+ DHA) concentrations (R^2^ = 0.70, p < 0.0001) (Fig. 3). Total lipids are therefore broadly predictive of omega-3 levels across species, though the relationship is less apparent within species or between species at low lipid concentrations. The relationship between total lipids and FA characteristics in fish tissues has been reported previously (Kainz et al., 2017), but has not been seen consistently across studies (Belling et al., 1997; Huynh and Kitts, 2009). Levels of omega-3 FA’s from a serving (227 g) of fish meet the level recommended (500 mg daily intake) (USDA and HHS, 2020) for the oily fish species, dogfish and herring.

**Fig. 4.**
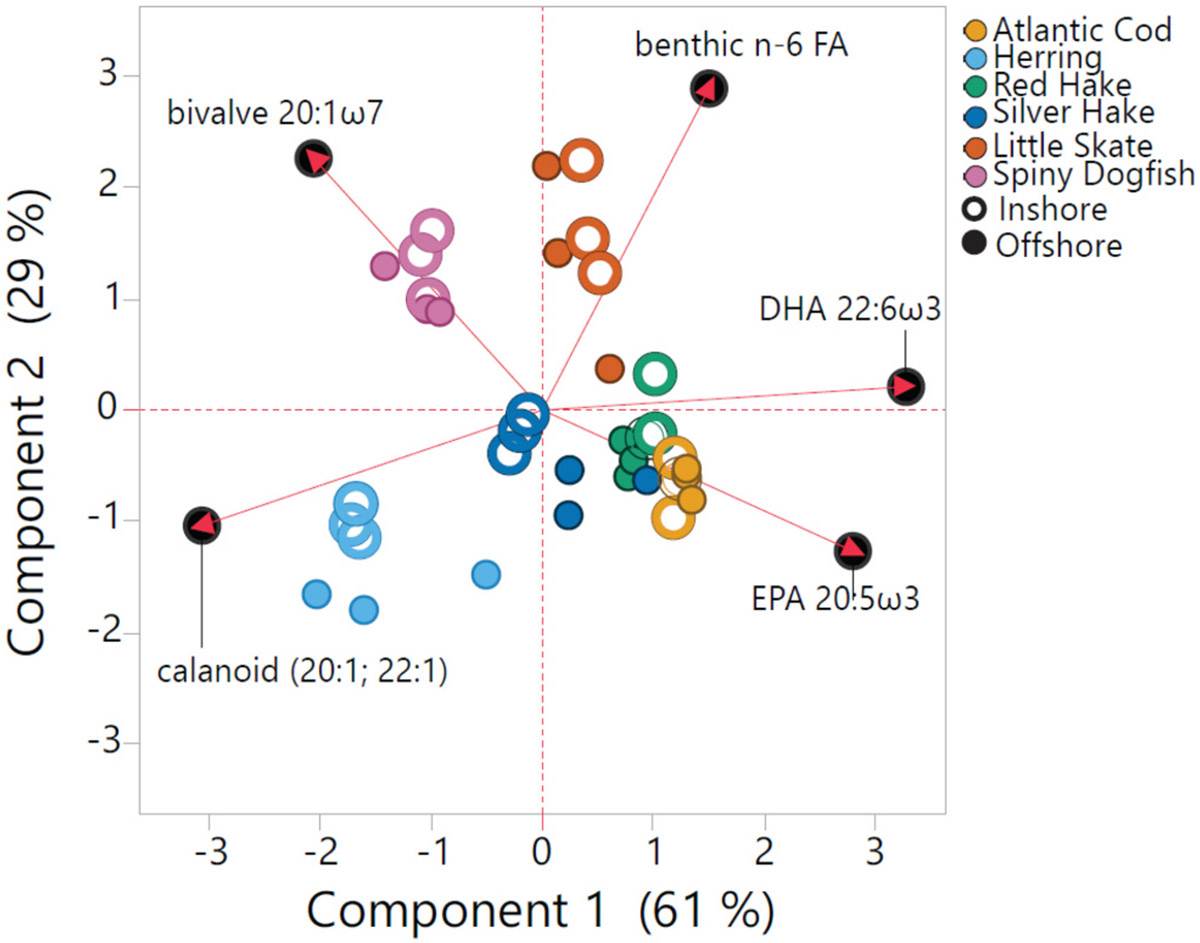
Principal component analysis of select fatty acid biomarkers across fish species (all locations combined).

Stable isotopes (SI) and FA profiles were also examined to investigate food sources across and within species (Tables 1S, 4S). Values of δ^15^N are an indicator of trophic position (Peterson and Fry, 1987), but in coastal ecosystems inputs of nutrients can also enrich ^15^N at the base of the food web (McClelland et al., 1997; Vizzini and Mazzola, 2006; Ẑvab Rožič et al., 2014). Higher δ^15^N were observed in inshore vs. offshore fish of all species except dogfish (*p* < 0.05) which is likely attributed to coastal inputs of nutrients, which confound interpretation of trophic level. In marine fish, δ^13^C, which reflects food sources (Peterson and Fry, 1987), is a broad indicator of benthic vs. pelagic production (e.g. Stribling and Cornwell, 1997), but can also be influenced by terrigenous sources of carbon in marine sediments which are higher in inshore waters resulting in more depleted δ^13^C values (Nagao et al., 2005) and by variations in plankton assemblages and growth rates (Goericke and Fry, 1994). The depleted δ^13^C ratios in herring and dogfish suggest more pelagic feeding, whereas enriched δ^13^C in skate reflect predominantly benthic food sources. However, the small range in δ^13^C across species in this study reflect a similar, phytoplankton base to the pelagic and demersal food webs in this region (Harding et al., 2018). Differences in food sources between species are evident in FA composition of fish tissues (Fig. 4). Marine lipids are derived predominantly from phytoplankton and are transferred to zooplankton and fish via pelagic food webs. The FA composition of fish reflected expected food sources, with the benthic markers n-6 FA most prominent in skate, the bivalve marker 20:1ω7, overlapping with the spiny dogfish, and the calanoid zooplankton marker pronounced in herring. EPA and DHA (as % of total FA) were most prominent in lean white fish (silver and red hake, cod) (Copeman and Parrish, 2004) with both δ^13^C (Fig. 2S) and FA composition (Fig. 4) suggesting dietary overlap in these species.

### 3.2. Drivers of Hg and Se bioaccumulation, and lipid content

Bioaccumulation of Hg in coastal fish have been found to vary across species broadly based on their habitat use (Azad et al., 2019; Sulimanec Grgec et al., 2020), size, age (Harding et al., 2018; Taylor et al., 2014) and trophic level (Cyr et al., 2019; Karimi et al., 2014; Mauffret et al., 2023). Here, a multivariate model found habitat (demersal, benthopelagic, pelagic) and length explained 73 % of the variation in fish Hg concentration (*p* < 0.0001, Table 5S) across all species, with cod and dogfish, the largest species, having the highest Hg. A previous study of Hg in food webs in the Bay of Fundy found fish size and trophic level, which were closely related, to be the major drivers of Hg bioaccumulation, but that Hg concentrations across species were not clearly differentiated by habitat (Harding et al., 2018). In the current study, trophic level determined by δ^15^N was not strongly correlated with log_10_Hg concentrations across all species (Fig. 4S), in part due to the similar trophic levels of these fish, and the variation in δ^15^N within species due nutrient inputs. Other coastal studies have found Hg concentrations to vary across species by habitat in the order: demersal > benthopelagic > pelagic (Azad et al., 2019; Sulimanec Grgec et al., 2020). In this study, there was large variation in Hg within species, and overlap among habitats, evidenced by the narrow range of δ^13^C ratios; this was attributed to a similar planktonic base to the food web across habitats in this well mixed system (Harding et al., 2018). Across habitats, the most pelagic species, herring and the most demersal, skate, had similar concentrations of Hg. The large variation in Hg bioaccumulation within species is discussed below.

Accumulation of Se was similar across all species with the exception of skate (Fig. 2), and was only related to habitat, whereas length, SI and location were not correlated to Se across all species (p < 0.0001, Table 5S). The low variance of Se across species has been observed in other studies of marine fish in the northeastern U.S. (Burger and Gochfeld, 2021, 2013, 2011; Karimi et al., 2013), and attributed to regulated uptake of this essential nutrient (Burger and Gochfeld, 2011). The higher Se values in skate may be explained by their feeding on benthic invertebrates, which accumulate Se more efficiently than pelagic food sources such as plankton (Presser and Luoma, 2010; Stewart et al., 2004). The negative correlation between Hg concentration and Se:Hg molar ratio is expected due to the much higher variability in Hg than Se concentrations. These trends are similar to those observed in other surveys of Hg and Se (Azad et al., 2019; Burger and Gochfeld, 2021, 2013, 2011).

Lipid content varies by habitat, with size, and ontogenetic and potentially seasonal changes in diet also influencing fat content. Across all species, lipid content was related to multiple factors (habitat, size, location, δ^13^C, δ^15^N; Table 5S). Herring, which are pelagic planktivores, and dogfish, which are large predators, as expected had the highest lipid content, as well as the highest concentrations of ω3 (Pateiroa et al., 2022). Across seasons and locations, variation in phytoplankton community composition and density influences ω3 and lipid concentrations in plankton (Bi et al., 2020; Hixson and Arts, 2016), which may be transferred to higher trophic level organisms.

### 3.3. Concentrations of Hg, Se, and lipids within fish species: effects of fish length and location

**Fig. 5.**
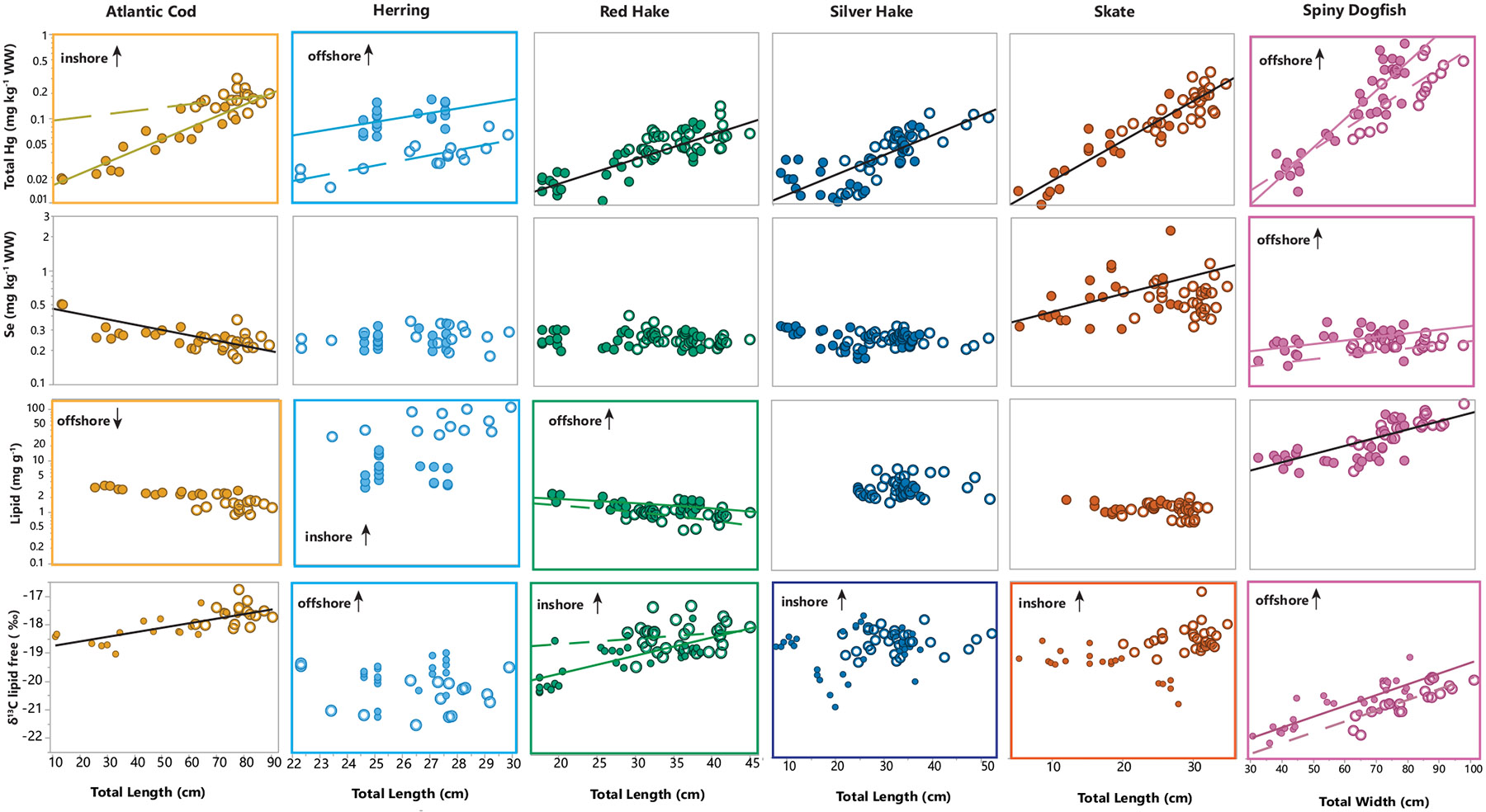
Concentrations of Hg (mg kg^−1^ wet weight), Se (mg kg^−1^ wet weight), total lipid (mg/g wet weight) and ^13^δC vs. length in fish from the Gulf of Maine. Fish from the offshore regions are symbolized by solid circles and those from inshore regions are symbolized by hollow circles. Significant relationships (p < 0.05) between concentration and length are denoted by a line (solid for offshore and dashed for inshore). Significant differences (p < 0.05) between concentrations in fish caught inshore and offshore are denoted by a colored frame around the plot, and the location with the higher concentrations labelled on the plot. Note that total lipid values are missing for the smallest individual fish due to insufficient sample mass for analysis. Least squares models are provided in Supplemental Table 5S.

The effect of fish length and catch location (inshore vs. offshore) on total Hg, Se and lipid concentrations was explored for each species (Fig. 5; Table 6S), to better understand the spatial and life history drivers of contaminants and nutrients. Fish length was a driver of Hg concentration for all species of fish, which is expected due to the long biological half-life of Hg (Van Walleghem et al., 2013, 2007) driving higher Hg concentrations in older, larger fish. Unlike previous studies in which Se has not varied with marine fish size (Burger and Gochfeld, 2011), fish length was related to Se concentrations in cod, skate and spiny dogfish, though the relationship was positive for skate and dogfish, and negative for cod. Variation in Se within species was low, with the exception of skate. Total lipids increased with length in spiny dogfish, whereas there was a negative relationship between length and lipid concentrations for red hake.

Catch location influenced Hg concentration for cod, herring, and dogfish, though for herring and spiny dogfish, length-corrected means of Hg in offshore fish were highest, whereas for cod, inshore fish had higher concentrations of Hg when corrected for length (Fig. 5). Only dogfish displayed a catch location effect for Se concentrations, which were higher in offshore fish. Total lipids differed between inshore and offshore sites for cod, herring, and red hake, with higher lipid content in the offshore cod and hake, but in the inshore herring, which is opposite of the pattern observed in Hg levels. The lower Hg observed in offshore cod and red hake coincided with higher lipid concentration, together indicating a favorable health benefit in these fish, and following the predicted trend. For herring and dogfish, this pattern is reversed, where the inshore fish have lower Hg levels, and in herring, higher concentrations of lipids. For dogfish, offshore fish also had slightly higher length-adjusted mean Se levels, which are nutritionally beneficial.

Significant differences in δ^13^C ratio between inshore and offshore fish were observed for all species of fish except cod, suggesting different basal food sources in diets for the inshore and offshore fish. However, opposite patterns of δ^13^C were observed for herring and dogfish, which had more enriched δ^13^C in offshore fish than their inshore counterparts, whereas for other species, inshore fish were more enriched in δ^13^C; (Fig. 5). The enriched δ^13^C in offshore pelagic fish therefore coincided with higher Hg and lower lipids in these species.

### 3.4. Ecological drivers of Hg, Se, and lipid concentrations in fish

The high Hg levels in offshore fish occurred in the two most pelagic species, Atlantic herring and spiny dogfish, though they vary greatly in size, feeding behavior, and taxonomic class: Atlantic herring are small bony fish (Osteichthyes) that feed predominantly on zooplankton, and spiny dogfish are much larger, cartilaginous fish (Chondrichthyes) that are predatory and opportunistic feeders. For Atlantic cod, which are demersal and similar in size to dogfish, Hg concentrations were higher inshore, which was expected for all species due to the river inputs of Hg to the inshore environment. The contrasting spatial patterns in Hg loading may be driven by several factors. For cod, it is noted that size distributions were different between inshore and offshore fish, where the larger size of the inshore cod (Fig. 5, Table 6S) may have had some influence on their higher least-squares mean Hg concentration (crosseffect for length*location: *p* = 0.054). Size was not a confounding factor for locational differences in lipids for cod, which were higher offshore, but was correlated with C:N ratio, an alternate proxy for lipid content (Fig. 6S).

The different catch timing likely played a role in the patterns of Hg, lipids and δ^13^C in the inshore and offshore fish. Inshore fish were captured in May/June, whereas offshore fish were caught in October/November, such that differences in Hg, lipids and δ^13^C, reflect different stages of spawning, migration and seasonal feeding habits. For herring, there are several different stocks within the Gulf of Maine, which spawn in coastal areas in the fall where they over-winter and move offshore in spring to feed (Tobin-Van Den Heuvel et al., 2022). Cod also inhabit the deep offshore waters during the spring and summer and move to shallower waters in the winter. Spiny dogfish inhabit the GOM year-round, though some fish move further offshore during the winter. Higher food abundance and warmer temperatures in the inshore waters in spring may support rapid growth in the inshore herring, which may also contribute to lower Hg levels via growth dilution (Cossa et al., 2012; Karimi et al., 2016). Considerable variation in lipid content (~10 to 200 mg/g) was also observed in a large survey of herring from the Gulf of Maine, and fish caught in the summer were fatter than those caught in winter (Lane et al., 2002). Spawning has also been associated with a decline in lipid content (Huynh et al., 2007), which may contribute to the lower lipid concentrations in herring in spring. By contrast, a study of cod suggested a decline in fat content from fillet following spawning in spring (Dambergs, 1964), though another study found no seasonal trend (Jangaard et al., 1967). Fish which have migrated offshore to colder waters, may have higher metabolic activity and slower growth, whereas those feeding inshore may be growing rapidly, leading to growth dilution of Hg in fish tissues (Cossa et al., 2012). Growth dilution is expected to have a larger effect on small pelagic fish than larger demersal species, contributing to the opposing patterns of lower Hg in inshore pelagic fish, compared to higher Hg in the demersal species.

Locational differences in fish Hg may also be driven by marine MeHg levels and bioavailability. Riverine waters typically have higher Hg concentrations than marine waters, but MeHg levels are less predictable and may be higher offshore. In addition, estuaries are associated with higher levels of terrestrial organic matter, which lower MeHg bioavailability to the pelagic food web (Chen et al., 2014; Taylor et al., 2019).

The different inshore-offshore patterns of δ^13^C between species suggest differences in basal food sources between these locations and/or seasons related to catch timing. Herring and dogfish have the most depleted δ^13^C ratios in the study (Fig. 3S), which is typical of pelagic feeders (Peterson and Fry, 1987), however the pattern of higher δ^13^C ratios in offshore herring is unusual (Fig. 5). Other studies in the region have found variable δ^13^C ratios in herring: in the Bay of Fundy, herring had more depleted δ^13^C ratios (−21.0 ± 0.8, *n* = 6 (Harding et al., 2018)) than those in this study (offshore: −19.1 ± 0.7, *n* = 19; inshore: −20.0 ± 0.7, *n* = 16). However, a study of herring across the continental shelf of the NW Atlantic (including the Gulf of Maine, George’s Bank and the Middle Atlantic Bight) found more enriched δ^13^C ratios (−18.5 ± 0.6, *n* = 126). In that study, δ^13^C ratios varied by season but not location, and were more enriched in spring, which is the opposite pattern of this study. The seasonal pattern coincided with small copepods being an important component of herring diet in the spring, and krill and hyperiid amphipods dominating diet in the fall (Suca et al., 2018). Other studies have also found krill to dominate Atlantic herring diets in the Gulf of Maine and Gulf of St. Lawrence (Bowman, 2000; Hanson, 2018).

Differences in microplankton assemblage between sites/seasons may explain the pattern in δ^13^C in herring, and potentially dogfish, which feed on herring, in this study. Previous studies in the Gulf of Maine have found that small, diatom-rich plankton fractions had enriched δ^13^C (−18.4 ± 0.9 (Harding et al., 2018); −19.4 to −15.5 ‰ (Fry and Wainright, 1991)), and provide a source of δ^13^C-rich carbon to marine food webs (Fry and Wainright, 1991). Diatom blooms vary spatially and temporally, and occur most intensely in offshore waters in early spring, with a second bloom in late fall (Kane, 2011; Thomas et al., 2003). Changes in δ^13^C in herring may be driven by diatom blooms, whereas a shift in food source from copepods to krill may not be reflected in δ^13^C ratios in the Gulf of Maine, as Harding et al. (2018) found mesoplankton, dominated by copepods, and nekton, dominated by krill, had similar δ^13^C. A shift in food source may influence Hg and lipid concentrations, which are also attributed to trophic level and prey assemblage (Taylor et al., 2014). In the Bay of Fundy, higher MeHg and Hg concentrations were found in the larger, higher trophic level nekton than in the copepods (Harding et al., 2018). A seasonal switch from copepods to krill may therefore result in higher MeHg bioaccumulation in pelagic fish. The impact of a seasonal change in feeding on lipid content in herring is unclear (Suca et al., 2018), however, Atlantic herring were found to prey selectively on calanoid copepods in the spring despite higher abundances of krill at that time, but consume krill and hyperiid amphipods in the fall during the absence of calanoid copepods, suggesting they are less well suited to a diet of krill (Suca et al., 2018). The absence of the preferred lipid-rich copepod prey in fall may contribute to the lower lipid contents in the offshore herring.

The inshore/offshore difference in Hg in dogfish, which are omnivorous and feed primarily on pelagic squid and bony fish, including herring (Taylor et al., 2014), may also be driven by both spatial and temporal variation in these prey species, though short-term seasonal differences in Hg concentration are less likely to be evident in these larger, long-lived fish (Van Walleghem et al., 2013). Differences in basal food source between inshore and offshore dogfish were not evident in δ^13^C ratios. However, locational differences in Hg concentrations in spiny dogfish have been reported previously (St. Gelais and Costa-Pierce, 2016; Taylor et al., 2014). Concentrations of Hg in this study were higher than a previous study of the Gulf of Maine (St. Gelais and Costa-Pierce, 2016), but in the same range as those from other studies in the Northeast (Maynard and Baumann, 2020; Taylor et al., 2014). The variation in Hg between onshore and offshore dogfish may reflect Hg loading in prey, which include herring.

The Gulf of Maine is undergoing rapid environmental change, and the patterns in Hg and lipids are likely to be influenced by these perturbations. Large shifts in plankton community and density have been observed over the past several decades, particularly a decline in calanoid copepods (Pershing and Kemberling, 2024) and an overall decrease in phytoplankton density (Balch et al., 2022). These changes are likely to influence preferred prey availability to the food web, and influence both lipid content and Hg bioaccumulation, particularly in pelagic fish. Increased river runoff and associated organic matter inputs (Balch et al., 2016; Huntington et al., 2016) may also drive higher Hg inputs but lower Hg bioavailability in the nearshore regions of the Gulf. The unexpected patterns of Hg and lipids observed in fish from the Gulf of Maine, with higher Hg and lower lipids found in offshore pelagic fish, and the opposing pattern in demersal fish, suggest different susceptibilities of fish to Hg bioaccumulation and nutritional status based on their food sources.

## 4. Conclusions

Concentrations of Hg, Se, and lipids measured concurrently in commercially important marine fish species from the Gulf of Maine show variation in Hg and lipid concentrations between species was driven by size and trophic level rather than habitat, whereas Se only differed in skate, the most benthic species. Within species, Hg was strongly related to fish length, as were Se and lipids in a few species. There were important differences in inshore versus offshore catch locations for concentrations of Hg and lipids, which we hypothesize are driven by differences in prey preference and availability, and for Hg, potential differences in bioavailability. We note that organic contaminants, particularly perfluor-alkyl contaminants, which were not included in this study, have the potential to alter fish consumption advice as they accumulate via different pathways than Hg (Wu et al., 2025). Information presented here on Hg and nutrients measured in fish across a range of fisheries species is valuable for assessing the risks and benefits of seafood consumption.

## Supplementary Material

SI_2

SI_1

Supplementary data to this article can be found online at https://doi.org/10.1016/j.scitotenv.2025.179786.

## Data Availability

Data is presented in Supplemental Files.

## HIGHLIGHTS

- Patterns of mercury, selenium and lipid concentrations in commercial coastal fish
- Fish mercury levels across species influenced by size and life history over habitat
- Mercury and lipid spatial patterns differ for pelagic and demersal fish
