# Supplementary material for: Geographic and ecological drivers of contaminants and nutrients in commercial fish species
Source: Sci Total Environ. Author manuscript; Available in PMC 2026 Apr 29. (PMC12334946; doi:10.1016/j.scitotenv.2025.179786)
Supplement: SI_1 [file NIHMS2096158-supplement-SI_1.docx]

**Geographic and ecological drivers of consumption risk-benefit in commercial fish species**

V.F. Taylor, K.L. Buckman, C.Y. Chen

Figure 1S: MeHg (mg kg^-1^) vs. Total Hg (mg kg^-1^) in a subset of 12 fish from each of six species (6 from inshore and 6 from offshore locations).

Figure 2S: Molar ratio of Se:Hg vs. concentration of Hg (mg kg^-1^ WW).

Figure 3S: δ^13^C vs. δ^15^N ratios for six species of fish from the Gulf of Maine.

Figure 4S: log_10_ Hg (mg kg^-1^) vs. δ^15^N ratios for six species of fish from the Gulf of Maine. Correlation coefficients (R^2^) are given for each fish species.

Figure 5S. Concentrations of C:N ratio in six fish species (all locations) from the Gulf of Maine.

Figure 6S. C:N ratio in six fish species (all locations) vs. length in fish from the Gulf of Maine. Fish from the offshore regions are symbolized by solid circles and those from inshore regions are symbolized by hollow circles. Significant relationships (p < 0.05) between concentration and length are denoted by a line (solid for offshore and dashed for inshore). Significant differences (p < 0.05) between concentrations in fish caught inshore and offshore are denoted by a colored frame around the plot, and the location with the higher concentrations labelled on the plot. Note y-axes change between plots. Least squares models are provided in Supplemental Table 6S.
